# Polypyrrole-Modified *Saccharomyces cerevisiae* Used in Microbial Fuel Cell

**DOI:** 10.3390/bios15080519

**Published:** 2025-08-09

**Authors:** Kasparas Kižys, Domas Pirštelis, Ingrida Bružaitė, Inga Morkvėnaitė

**Affiliations:** Department of Nanotechnology, State Research Institute Center for Physical Sciences and Technology, Sauletekio 3, LT-10257 Vilnius, Lithuania

**Keywords:** *Saccharomyces cerevisiae*, polypyrrole, 9,10-phenanthrenequinone, microbial fuel cells, electrochemistry

## Abstract

Microbial fuel cells (MFCs) are one of the contributors to the novel sustainable energy generation from organic waste. However, the application of MFCs is limited due to the slow charge transfer between cells and electrodes. This problem can be solved by modifying cells with conductive polymers, such as polypyrrole (PPy). We investigated the viability and electroactivity of modified cells at five different pyrrole concentrations, namely 8, 25, 50, 100, and 200 mM. The 100 mM concentration of PPy solution had the highest impact on yeast cells’ proliferation and growth, with the CFU/mL of PPy-treated yeast cells being 0.6 × 10^7^ ± 5 × 10^−2^. The power density of the constructed MFC was evaluated by using an external load. The MFCs were analyzed using cyclic voltammetry (CV) and differential pulse voltammetry (DPV). Although CV results with different pyrrole concentrations were similar, DPV indicated that yeast modification with 50 mM pyrrole resulted in the most significant current density, which may be attributed to an increase in charge transfer due to the conductive properties of polypyrrole. The power density achieved with modified yeast in wastewater, 12 mW/m^2^, reached levels similar to those in laboratory solutions, 45 mW/m^2^.

## 1. Introduction

In industrial wastewater facilities and research communities, interest in introducing green energy solutions from wastewater, biofuel, or biomass sludge is growing rapidly [1,2]. To address such an issue, biofuel cells can be utilized, involving a bioelectrochemical method that generates electrical current from waste. When a microbe is used for power generation, biofuel cells are referred to as microbial fuel cells (MFCs) [3,4]. The bioconversion of waste-derived saccharides into electrical energy critically depends on the use of diverse microbial species, including yeast *Saccharomyces cerevisiae* [5,6,7]. Their metabolic plasticity, resilience in fluctuating effluent conditions, and well-established roles in biotechnological applications make them strong candidates for sustainable bioelectricity production [8]. Wastewater as a viable fuel source is used only after the pasteurization of the sludge to ensure that the local bacterial and pathogenic organisms are not present. MFC can be used for pollutant removal with energy recovery from wastewater [8,9,10].

The use of biocompatible and conductive substances, such as nanoparticles, polymers, and polypyrrole, has the potential to enhance charge transfer between the microorganism and the electrode. Conductive polymers can modify the yeast cell wall to increase electrical conductivity and improve charge transfer. Among others, polypyrrole (PPy) has the highest biocompatibility with living cells and microorganisms [11]. PPy used together with gold nanoparticles in MFC enhances the power density three times compared to unmodified cells [12]. Previous studies have shown that polypyrrole (PPy) forms predominantly on the outer surface of yeast cells, particularly within the cell wall structure, rather than penetrating the plasma membrane. Andriukonis et al. demonstrated that PPy synthesized in the presence of *Saccharomyces cerevisiae* binds to cell wall components, influencing mechanical properties without directly disrupting internal organelles [13,14]. Similarly, Zinovicius et al. confirmed that PPy deposition occurs on the yeast wall matrix, enhancing surface conductivity but posing potential barriers to mass transport at higher polymer loadings [15]. These findings support the hypothesis that excessive PPy can physically encapsulate cells or trigger stress responses, thereby reducing viability even if internal cell membranes remain intact.

However, the application of conductive polymers such as polypyrrole (PPy) in microbial fuel cells is limited by their dual effect on microbial systems. While PPy enhances electron transfer by increasing cell surface conductivity, high concentrations can induce oxidative stress, interfere with cell wall integrity, and hinder nutrient exchange—ultimately reducing microbial viability. To address this challenge, it is essential to identify an optimal concentration range that balances improved electroactivity with minimal cytotoxic effects.

While microbial fuel cells (MFCs) have shown promising results in controlled laboratory media, their real-world application in municipal wastewater environments remains challenging. Wastewater presents a complex and variable chemical composition, including organic matter, micronutrients, and potential inhibitory substances, making it both an opportunity and a challenge for MFC development [16,17]. Conductive polymer-based microbial systems, though effective in ideal lab conditions, can exhibit reduced performance or instability in real wastewater due to factors like cytotoxicity, substrate limitations, and fouling. In our previous research, we showed that MFC in a wastewater sample yielded a power density three times higher than in the laboratory solution [12]. However, excessively high polymer concentrations can inhibit microbial viability, particularly in such nutrient-variable systems. Therefore, there is a need to identify an optimal modification strategy that enhances electron transfer while maintaining microbial activity in realistic wastewater matrices. This study aims to address that gap by evaluating the viability and electrochemical performance of *Saccharomyces cerevisiae* modified with various concentrations of polypyrrole in both laboratory and wastewater conditions, helping to establish design guidelines for practical, scalable MFCs.

This study aims to evaluate the impact of polypyrrole concentration on the viability and electroactivity of *S. cerevisiae* in MFCs, with the goal of optimizing performance in wastewater applications.

## 2. Materials and Methods

### 2.1. Materials

High-purity materials (>99%), such as potassium (II) ferricyanide, potassium (III) ferrocyanide, 9,10-phenantrenequinone, YPD media, agar, pyrrole, D-(+)-glucose, and phosphate-buffered saline (PBS) tablets were purchased from Merck (Darmstadt, Germany). Cyclopore Track Etched Membrane was purchased from Whatman^®^. Yeast cell strain *S. cerevisiae* Y10000 (BY4741 Matαhis 3Δ1 leu 2Δ0 met 15Δ0 ura 3Δ0) was purchased from EUROSCARF (Frankfurt, Germany). Graphite rod electrodes (99.9995%) were obtained from Thermo Fisher Scientific Baltics (Vilnius, Lithuania).

### 2.2. Methods

#### 2.2.1. Yeast Sample Preparation

*S. cerevisiae*, set Y10001 culture, was prepared in a laminar flow environment and in a temperature-controlled incubation cabinet. The yeast cultures were inoculated onto 5% YPD-agar Petri dishes using a deep-frozen library sample; the samples were then cultivated in a +30 °C incubator for 48 h. For further preparation of yeast cells, autoclaved 5% YPD broth vials were inoculated using the grown colonies, and cultivation was proceeded for an additional 24 h in a 30 °C incubator, shaking at 200 revolutions per minute. After the YPD broth vials were fully inoculated, the media with the cells was transferred into 1.5 mL Eppendorf vials for the separation and washing of yeast cultures by using a centrifuge, spinning at 5500 RPM for 5 min. The supernatant was discarded, and the samples were replaced with a PBS solution. The samples were then homogenized and centrifuged for an additional 5 min. This procedure was performed a total of three times. After discarding the remaining supernatant, the pellet was weighed and diluted with PBS to obtain a 1 g/mL yeast solution. The mixture was homogenized, and the resulting suspension was used for further yeast cell modifications or construction.

The modification of yeast cells with pyrrole was performed as described in [12]. Five different pyrrole concentrations were used—8, 25, 50, 100, and 200 mM. After the modification, a yeast in solution in PBS was prepared as described previously.

#### 2.2.2. Investigation of Viability of Polypyrrole-Modified Yeast

A 100 µL yeast suspension was diluted with PBS buffer solution to achieve a concentration of 10^5^–10^6^ cells per solution. A total of 100 µL of the diluted yeast suspension was plated onto agar-solidified YPD medium. Petri dishes were incubated in a thermostat at 30 °C for 48 h. Colonies were counted, and the number of colony-forming units (CFUs) per milliliter was determined using the following formula:

(1) $$CFU=\frac{A\cdot 10^{n}}{V}$$

where *CFU* is the number of viable yeast per 1 mL; *A* is the number of colonies on the plate; 10*^n^* is the dilution factor; and *V* is the volume of diluted sample plated on the agar in mL.

#### 2.2.3. Construction of Graphite Electrodes with Yeast Modification

**Figure 1:**
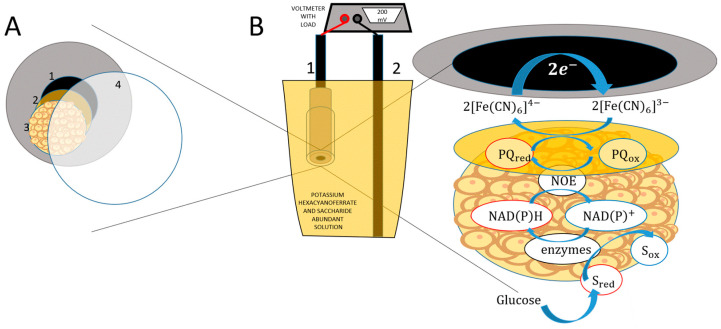
Schematic representation of the MFC and charge transfer mechanism. (**A**). 1 is graphite, 2 is PQ, 3 is yeast, 4 is membrane. (**B**). 1 is anode, 2 is cathode.

The modification scheme is shown in Figure 1. Graphite rod electrodes with a diameter of 3.05 mm were cut into 30 mm long strips, which were polished using sanding papers with different grit sizes ranging from 300 to 10,000. Furthermore, the electrodes were washed with methanol and deionized water, and a silicon tube with a diameter of 3.00 mm was fitted onto the polished end of the electrode.

A 3 mM solution of 9,10-phenantrenequinone was prepared in 96% ethanol; 4 µL of the mediator solution was dropped onto the polished end of the graphite electrode. After the solvent had evaporated, 2 µL of the yeast cell solution was applied to the mediator-modified surface. Lastly, the electrode surface was covered with a membrane and held in place with laboratory tape. The prepared electrode was submerged in PBS.

#### 2.2.4. Voltammetry Measurements

For the cyclic voltammetry and differential pulse voltammetry measurements, a three-electrode electrochemical cell was used, consisting of a yeast- and mediator-modified graphite working electrode, an Ag/AgCl reference electrode (3M KCl), and a platinum rod counter electrode (Metrohm AG, Switzerland). A solution containing 50 mM of glucose and 20 mM of potassium hexacyanoferrate (III) was used for the measurements inside the electrochemical cell. All electrochemical measurements were conducted using a potentiostat Autolab PGSTAT302N (Metrohm AG, Switzerland).

#### 2.2.5. MFC Measurements of Power Density

For the power density measurements, a single-chamber MFC was constructed, consisting of a yeast- and mediator-modified graphite anode and a graphite cathode (the surface of the cathode exceeded that of the anode by at least 10 times) submerged in a solution in a beaker. The experiments were conducted in either a laboratory medium consisting of 20 mM K_3_[Fe(CN)_6_] and 50 mM glucose in PBS or municipal wastewater samples provided by UAB “Vilniaus Vandenys”, a wastewater treatment facility. This wastewater sample consisted of thermally sanitized and laboratory-diluted sludge from the facility. It had been concentrated in UAB “Vilniaus Vandenys” from actual wastewater, which was then fermented and autoclaved during the process and subsequently distributed to our laboratory for dilution. It contained no living microorganisms and had a pH range of 7.6–7.8. COD as well as BOD were not determined in either UAB “Vilniaus Vandenys” or in our laboratory due to the non-identifiable nature of concentrated wastewater in the sludge; this sample was used to represent the principle of our MFC, not to determine the capability in real conditions. The sample was used only for experimentation over a 45-day period and was divided into dilution and repetition of power density trials. Before measurements, the working electrode was left in the solution for 20 min for incubation.

The power density of the constructed MFC was evaluated by using an external load, with resistances ranging from 1000 kΩ to 100 Ω (for wastewater samples) or from 1000 kΩ to 1 Ω (for laboratory samples). Voltage was measured during the connection of the external load and at one-minute intervals for a total of 3 min.

## 3. Results

### 3.1. Evaluation of Yeast Viability

To evaluate the impact of PPy modification on the growth and proliferation of *Saccharomyces cerevisiae* yeast, colony-forming unit (CFU) assays were performed using PPy solutions at varying concentrations. Colony-forming unit (CFU) quantifications are presented in Table 1.

The provided bar chart demonstrates the effect of polypyrrole modification on the colony formation of *Saccharomyces cerevisiae* yeast on agarized YPD medium. The 100 mM concentration of polypyrrole solution had the most significant impact on yeast cells’ proliferation and growth, with the CFU/mL of polypyrrole-treated yeast cells being (0.6 ± 0.1) × 10^7^. This result might be indicating that the highest concentration given in the study is likely not suitable for use with living organisms due to cytotoxic effects.

**Figure 2:**
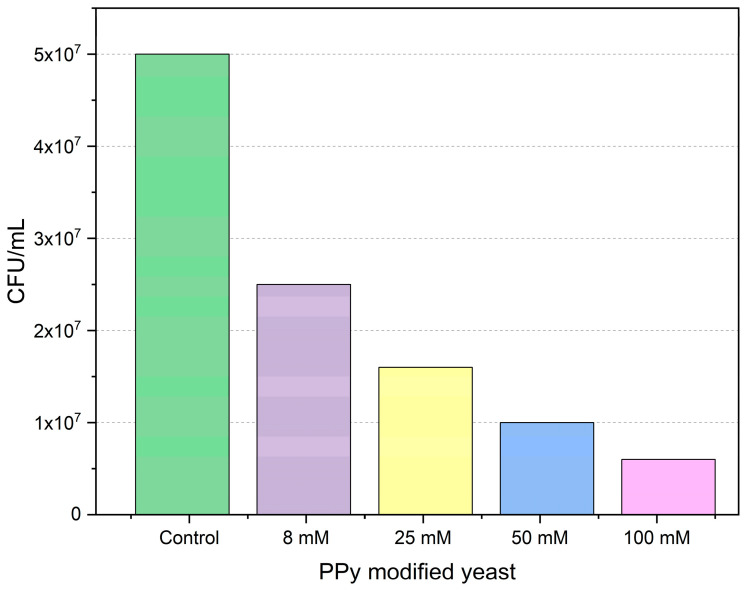
Effect of concentration of polypyrrole on the viability of *Saccharomyces cerevisiae* yeast cells. The results after several measurements have shown only marginal errors, which are not visible in the given graph.

Figure 2 illustrates the inhibitory effect of polypyrrole solutions on colony formation by yeast cells. In comparison to the control, all polypyrrole concentrations applied in the study significantly influenced yeast cell proliferation. Increased polypyrrole concentrations may have caused cellular stress in yeast, including oxidative stress and pH imbalance, which negatively affected cell growth and proliferation.

### 3.2. Voltammetry Measurements of MFC

**Figure 3:**
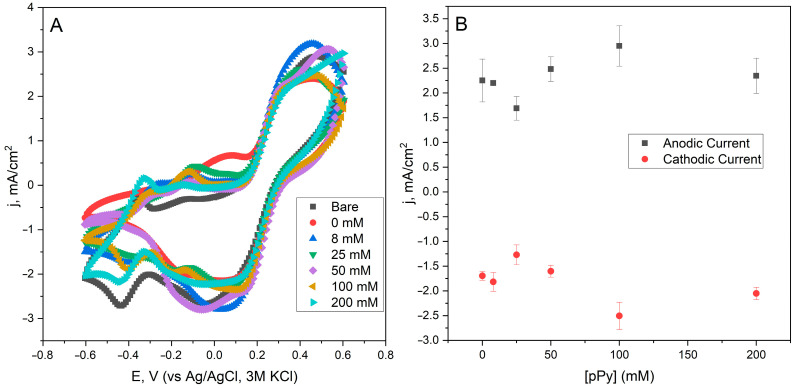
(**A**) Cyclic voltammetric curves of different PQ and modified yeast cell electrodes. Measurements were conducted by sweeping the potential from −0.6 V to +0.6 V at 50 mV/s using a Ag/AgCl reference electrode in KCl 3M. Bare: PQ-modified graphite electrode without yeast cells; 0 mM: PQ-modified graphite electrode with unmodified yeast cells; 8 to 200 mM: concentration of pyrrole used in modifying yeast cells that are used in the construction of the electrode. (**B**) Current density registered for oxidative (0.32 V) and reductive (0.11 V) potentials of the ferro/ferric redox couple for different concentrations of pyrrole used in the modification of yeast cells.

For the determination of the effect of PPy on the modification of yeast cells, which are used in the construction of yeast and the PQ- modified electrode, cyclic voltammetry measurements were performed (Figure 3A). The observed peaks in the cyclic voltammetry curves result from the combined redox behavior of the Fe^3+^/Fe^2+^ redox couple (from potassium ferri/ferrocyanide) and PQ, which was used as a mediator on the electrode surface. Specifically, the oxidative and reductive peaks near +0.32 V and +0.11 V (vs. Ag/AgCl) are attributed to the Fe^2+^/Fe^3+^ redox transitions in the bulk electrolyte. Additionally, PQ contributes to redox activity at the electrode surface, typically around +0.1–0.2 V, where it facilitates extracellular electron transfer from the yeast-modified anode. The enhancement of current densities with increasing pyrrole concentrations indicates improved electron transfer efficiency due to the increased surface conductivity of yeast cells. However, at higher concentrations (e.g., 100–200 mM), microbial viability is reduced. The peak current density values of the ferri/ferro redox couple were plotted against the concentration of PPy used in the modification of the yeast cells (Figure 3B). The maximum current density of 2.949 mA/cm^2^ (for oxidative peak) and −2.504 mA/cm^2^ (for reductive peak) was observed when using 100 mM of pyrrole for the modification of yeast cells. Although 100 mM PPy yielded the highest current in voltammetry, power output was lower, likely due to reduced microbial metabolism. In contrast, 50 mM balanced conductivity and viability, making it the optimal concentration. Interestingly, yeast modified with 8 and 25 mM pyrrole showed slightly lower current densities than unmodified yeast, which may suggest that suboptimal polymer levels can interfere with electron transfer without significantly enhancing conductivity.

**Figure 4:**
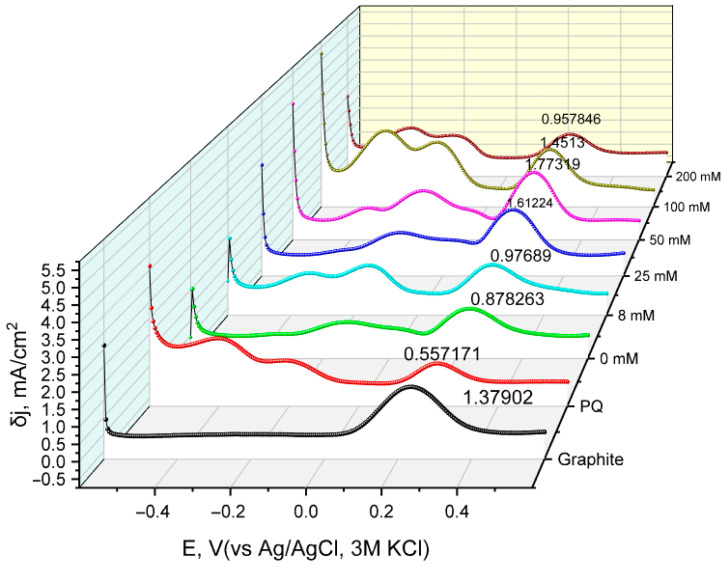
Differential current density curves for various modified working electrode samples used in the electrochemical system. Measurements were conducted by sweeping the potential from −0.6 V to +0.6 V with a step potential of 5 mV, pulse amplitude of 50 mV for a duration of 20 ms, and a scan rate of 50 mV/s using a Ag/AgCl reference electrode in KCl 3M. Graphite: unmodified graphite electrode; PQ: PQ-modified graphite electrode; 0 mM: non-modified yeast and PQ-modified graphite electrode; 8 to 200 mM: PQ- and PPy-modified yeast on graphite working electrodes.

In addition to CV measurements, differential pulse voltammetry curves were also obtained for the same constructed electrodes with varying pyrrole concentrations during the modification of yeast cells (Figure 4).

**Figure 5:**
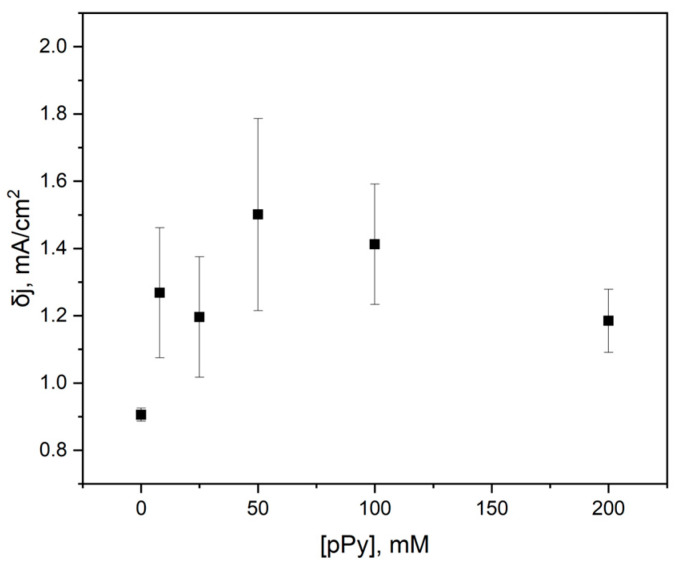
Differential current density dependency on the concentration of PPy used in the modification of yeast cells in the construction of the PQ-PPy yeast–graphite electrode.

The resulting Fe^2+^ oxidative peak δj values were plotted against the concentration of pyrrole used in yeast modification (Figure 5). The change in current density values indicates that using 50 mM pyrrole results in the most significant current density, which may be attributed to an increase in charge transfer due to the conductive properties of PPy.

### 3.3. MFC Power Density Measurements

**Figure 6:**
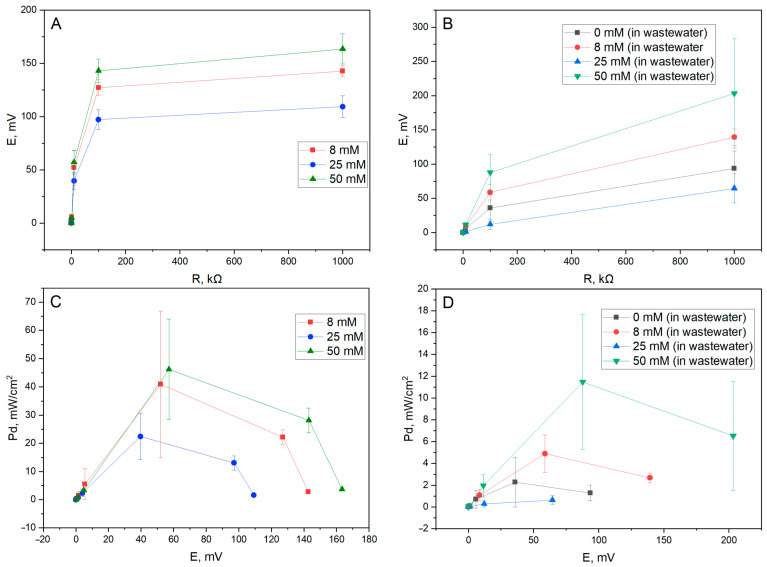
Power density measurements of the constructed yeast-based MFCs. (**A**) Potential load dependency of the MFC in 50 mM glucose and 20 mM K_3_[Fe(CN_6_)] solution in PBS while using yeast modified by different concentrations of PPy. (**B**) Potential load dependency of the system in wastewater samples for different PPy concentrations used in the modification of the yeast. (**C**) Power density potential dependency of the MFC in glucose/K_3_[Fe(CN_6_)] samples. (**D**) Power density potential dependency of the MFC in wastewater samples.

In addition to voltammetric methods, power density measurements were taken to determine the power output of our modified yeast-based MFC. A two-electrode, single-chamber MFC system was constructed utilizing our yeast-modified graphite electrode (anode) and a graphite cathode. Measurements were taken in either a glucose (50 mM) and K_3_[Fe(CN_6_)] (20 mM) solution in PBS (Figure 6A,C) or in municipal wastewater samples (Figure 6B,D).

The optimal concentration of PPy used for modifying the yeast cells in the context of the maximum potential/power output was determined to be 50 mM for both media samples. Nevertheless, a clear drop in both potential and power density values is visible when using wastewater media instead of a glucose and K_3_[Fe(CN_6_)] sample; this result may be attributed to a lack of a viable substrate for yeast metabolism and a lack of an optimal redox mediator in the wastewater solution. Additionally, a sharp decrease in power output when using 25 mM of PPy when modifying the yeast cells is again observed, as was observed during the voltametry measurements—this could indicate a cytotoxic or, in general, inhibitive effect this concentration may have on the energy generation from the microbes.

## 4. Discussion

This study highlights the impact of polypyrrole (PPy) modification on the viability and electroactivity of Saccharomyces cerevisiae for microbial fuel cell (MFC) applications. Our findings demonstrate that while PPy enhances extracellular charge transfer, its concentration critically influences both electrochemical performance and microbial viability, echoing earlier studies that highlight the delicate balance between biointerface engineering and biocompatibility.

Viability assays revealed a sharp decline in colony-forming unit (CFU) counts as PPy concentration increased. At 100 mM pyrrole, CFU values were approximately 12% of those of untreated yeast, confirming significant cytotoxic effects. This corresponds with findings in [14,15,18], which showed that PPy coats the cell wall, altering mechanical properties and limiting mass exchange at high concentrations. Consequently, although PPy enhances conductivity, it poses stress on cell physiology, limiting long-term activity in bioelectrochemical systems.

Voltammetric measurements showed an increase in redox peak currents with higher PPy concentrations, especially at 50–100 mM. However, this trend plateaued or reversed at 200 mM, suggesting that beyond a threshold, improved conductivity is offset by reduced biological activity. This effect is consistent with the literature on PPy- or PANI-based modifications, which often exhibit optimal performance at moderate polymer levels due to a balance between charge mobility and cellular health [15,19]. Interestingly, voltammetric data suggested that 100 mM PPy yielded the highest current density (2.949 mA/cm^2^) in the oxidative direction, yet this did not translate to a higher power output. Therefore, voltammetry captures instantaneous redox activity, but sustained power generation depends on microbial metabolism, which is suppressed at higher PPy levels. Thus, electrochemical enhancement alone is insufficient if cellular functionality is compromised—a concept increasingly recognized in bioelectrochemical interface design [20,21].

The MFC power output profiles further emphasize this trade-off. In glucose–ferricyanide buffer, the 50 mM PPy-modified yeast electrode achieved the highest power density (~45 mW/m^2^), while both lower (8–25 mM) and higher (100–200 mM) concentrations underperformed. Similar trends were observed in wastewater media, although power densities were reduced (~2–12 mW/m^2^), likely due to suboptimal substrate availability and a lack of additional mediators. These observations are consistent with known limitations in real wastewater environments, where variable composition and inhibitory compounds reduce MFC output compared to controlled laboratory systems [2,9,16].

Comparing our results to other polymer-based MFCs (Table 2), the yeast–PPy system reported here shows competitive performance at 50 mM PPy, achieving similar or superior power densities to S. cerevisiae electrodes modified with CNTs [22] or PEDOT:PSS [23,24]. However, many high-performance systems in the literature use complex materials (e.g., PEI–nanoparticle composites [25,26]) or operate under idealized conditions, whereas our system relies on simple graphite electrodes and non-purified wastewater. This underscores the practicality and scalability of our approach.

In conclusion, the results indicate that moderate PPy modification (50 mM) strikes the optimal balance between microbial viability and charge transfer enhancement for yeast-based MFCs. Excessive polymer loading impairs bioactivity despite favorable voltammetric profiles, a crucial consideration for real-world deployment. Future optimization should explore co-polymers or bioinspired conductive matrices that support both electron mobility and microbial health. Moreover, strategies such as sludge preconditioning, mediator supplementation, or surface topography tuning may improve wastewater MFC efficiency without sacrificing sustainability.

This study highlights both the potential and ongoing challenges of MFC technology. While enhanced performance results from developments in electrode materials and a better knowledge of charge transfer routes [27,28,29], practical deployment is still limited by biological and environmental variability [30,31]. Our findings highlight the intricate interaction between living systems and electrochemical interfaces, as even minor changes in pH, substrate concentration, or microbial community composition can lead to substantial variations in electrical output [32,33,34]. The findings of anode microorganism modifications by conductive polymers are described in Table 2.

**Table 2:** Comparison of conductive polymers used in the MFC anode microorganism modification and their performance. Abbreviations are provided below the table.

Anode	Anode Material/Electron Donor	Power Density, mW m^−2^	Ref.
Mixed culture on PANI-graphene	carbon cloth/acetate	884	[35]
Shewanella xiamenensis on BC-PANI	BC/glucose	179.4	[36]
Shewanella loihica on PANI and carbon nanotubes	APTES, ITO/sodium lactate	34.5	[37]
Mixed culture on PEDOT:PSS-TEG	carbon felt/glucose	82	[38]
Mixed culture on PEDOT:PSS	carbon veil/urine	68.7	[39]
Mixed culture on MgCoO2-PEDOT:PSS	nickel foam/wastewater	0.54	[25]
Saccharomyces cerevisiae on PEI and one of the QS molecules (phenylethanol, ryptophol, and tyrosol)	CF/glucose	159 * 156 135	[26]
Saccharomyces cerevisiae on PEI and CNTs	CNTs/glucose	344	[40]
Saccharomyces cerevisiae on PEI and AuNPs	CF/glucose	2771	[41]
Saccharomyces cerevisiae on PEI, with SDBS and FeMnNPs	CF/glucose	5838	[24]
Mixed culture on PPy	stainless steel/wastewater	1190.94	[27]
Mixed culture on PPy	CMC-CNT carbon brush/acetate	2970	[28]
S. Cerevisiae on PPy	graphite rod/glucose	47.12	[15]
S. Cerevisiae on PPy-AuNP’s	graphite rod/glucose and wastewater	61.1 179.2	[12]

* power densities for MFCs based on phenylethanol, ryptophol, and tyrosol, respectively. APTES—γ-aminopropyltriethoxysilane. CF—carbon felt. MWCNTs—multi-walled carbon nanotubes. NPs—nanoparticles. CNT—carbon nanotubes. BC—biocellulose. FeMnNP—iron–manganese nanoflowers. CMC—carbomethyl cellulose. PEI—polyethylenimine. PU—polyurethane. PQ—9,10-phenanthrenequinone. QS—quorum sensing. PANI—polyaniline. TEG—thermally expanded graphite. ITO—indium tin oxide. SDBS—sodium dodecyl benzene sulfonate. PEDOT:PSS—poly(3,4-ethylene dioxythiophene):poly(4-styrene sul-fonate).

## Figures and Tables

**Table 1:** Dependence of colony-forming unit (CFU) count on polypyrrole solution concentration.

Sample	CFU/mL
Yeast cells treated with an 8 mM concentration of polypyrrole solution	(2.5 ± 0.5) ×∙10^7^
Yeast cells treated with a 25 mM concentration of polypyrrole solution	(1.6 ± 0.3) ×∙10^7^
Yeast cells treated with a 50 mM concentration of polypyrrole solution	(1.0 ± 0.2) ×∙10^7^
Yeast cells treated with a 100 mM concentration of polypyrrole solution	(0.6 ± 0.1)∙× 10^7^
Untreated yeast cells (control)	(5.0 ± 1.0) ×∙10^7^

## Data Availability

Data will be available on request.
